# Trend in the nutritional status of children aged 2-7 years in Luoding city, China: A panel study from 2004 to 2013

**DOI:** 10.1371/journal.pone.0205163

**Published:** 2018-10-03

**Authors:** Xiao-Ying Yan, Qin Li, Bing-Xian Luo, Tian-Hui You, Hai-Jun Wang

**Affiliations:** 1 School of Nursing, Guangdong Pharmaceutical University, Guangzhou, China; 2 Department of Maternal and Child Health, School of Public Health, Peking University, Beijing, China; 3 Luoding City Maternal and Child Health Care Hospital, Yunfu, China; TNO, NETHERLANDS

## Abstract

To examine trends in the prevalence of wasting, stunting, overweight, and obesity among children in Luoding, a lower-middle-income city in southern China, we collected height, weight and other information on 65,908 pre-school children aged 2 to 7 years from 23 kindergartens, in which health examinations were conducted annually between 2004 and 2013. We used the growth standards of the World Health Organization (WHO) to calculate Z-scores for height and body mass index (BMI), and used the cut-offs recommended by WHO to define wasting, stunting, overweight, and obesity for each child. From 2004 to 2013, the prevalence of overweight increased from 3.70% to 7.27% and of obesity increased from 1.04% to 2.08%. Meanwhile, the prevalence of wasting decreased from 0.91% to 0.72% and of stunting decreased from 9.29% to 5.22%. These trends suggest there was still a double burden of nutritional status there. The nutritional interventions focusing on pre-school children should be comprehensively elaborated in lower-middle-income areas such as Luoding.

## Introduction

A recent multi-country study predicts that in 2025, the prevalence of obesity will reach 18% in males and 21% in females respectively [1]. One of the main disconcerting aspects of the global obesity epidemiology is the high prevalence of childhood overweight and obesity [1]. It is well documented that overweight in childhood life is highly related to the increased risk of adulthood obesity and unfavorable cardio-metabolic outcomes, which may result in serious health and economic consequences [2].

On the other hand, in 2011 there were 165 million children under 5 with stunting worldwide, while Asia and Africa have reached 95.8 and 56.3 million, respectively [3]. Researchers predict that global stunting prevalence of children will reach 21% by 2020 [4]. Stunting in early childhood is related to poor cognition, low educational performance, low adult income, decreased offspring birth weight, and increased risk of metabolic diseases such as obesity or diabetes [5, 6].

The overweight and stunting among children become tremendous challenges in public health worldwide [7, 8]. In addition, stunting and overweight can coexist within a country, a city even a community. Some middle-income countries (e.g., China, India, Vietnam) are suffering from incessantly increases in overweight and a relative higher prevalence of stunting at same time. The co-existence of undernutrition (e.g., underweight and stunting) and overnutrition (defined as a child being overweight or obese), which was defined as a phenomenon of “double burden” of malnutrition by some previous studies [5, 9–11], poses a significant challenge to public health in these countries.

From the 1980s, China has been suffering from epidemiological and demographic transitions [12]. Traditional diets and activity behaviors have been replaced by high-energy intake and sedentary lifestyles among Chinese, which contribute to an increase in childhood obesity [13, 14]. A study based on the China Health and Nutrition Survey reports that 10% of the pre-school children aged 3 to 6 years in China were overweight [10]. On the other hand, due to obvious economic disparities, nutritional insufficiencies persist among Chinese children from middle and western cities, small cities and numerous county-level cities. A study reported that approximately 21% of children aged 3 to 6 years were underweight and 4% were stunting in 9 provinces of China [10]. These results suggested that some areas of China are suffering from the double burden seriously [15–17].

Identifying the trend of early childhood nutritional status (both undernutrition and overnutrition) is critical for prevention of nutrition-related chronic diseases. However, most previous researches focused more on school-aged children, the evidences for pre-school children are inadequate, especially on long-term trends in prevalence of wasting, stunting, overweight, and obesity [11, 15, 16, 18]. Even more, there is very limited information about children in low- and lower-middle income regions [18, 19].

In this study, we aim to explore the trends in prevalence of undernutrition and overnutrition (e.g., wasting, stunting, overweight and obesity) among children aged 2 to 7 years from 23 kindergartens in Luoding city, China, by analyzing the annual health examination data between 2004 and 2013. As Luoding is a lower-middle-income city located in Guangdong, one of the prosperous provinces in China, its traditional economy might be impacted by the neighboring cities such as Guangzhou and Shenzhen obviously. The economy of Luoding is an epitome of the economic disparities in China, which provides a chance to estimate whether there exist a double burden of overnutrition and undernutrition or not in lower-middle-income areas of China. Findings from this study will provide epidemiological evidence from lower-middle-income areas, which is conducive to public health interventions.

## Methods

### Ethics approval

Members of the survey’s staff explained the purpose of the survey to the parents of children and ensured that each participant signed a verbal agreement. The study project was approved by the Medical Research Ethics Committee of the first affiliated hospital of Guangdong Pharmaceutical University (No. 2017071). All data were fully anonymized before we analyzed them.

### Study population

**Fig 1:**
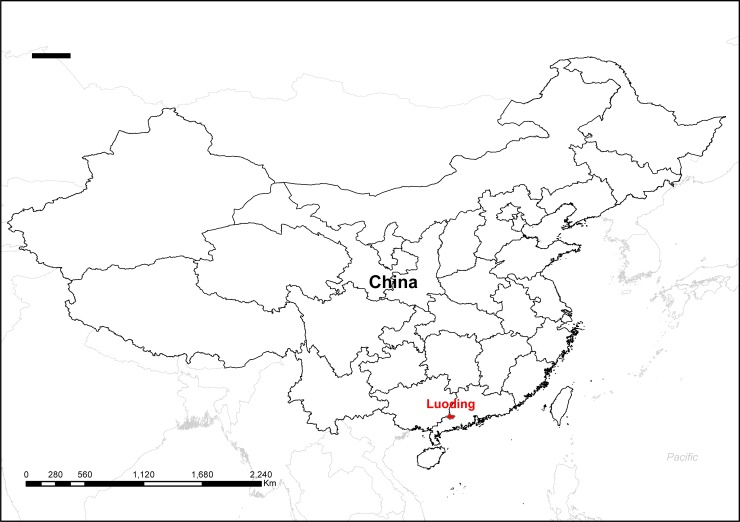
The geographic location of Luoding in China.

Luoding is a lower-middle-income (gross national income per capita between $1,006 and $3,955) city of China, with an area of 2327 km^2^ and a population of 1.25 million in 2013 (Fig 1). The demographic and socioeconomic characteristics of Luoding city from 2004 to 2013 were obtained from the Municipal Bureau of Statistics of Luoding. In general, local children aged 3 to 6 years go to kindergartens there. The Luoding Maternal and Child Health Care Hospital is responsible for promoting children’s health in the kindergartens. Health professionals from the hospital conduct health examination regularly for all children in kindergartens every year. In this study, we collected the children’s anthropometry data of the 23 kindergartens in Luoding that had ten years’ continuous data between 2004 and 2013. All the children in the study are aged from 2 to 7. Before measurement, a medical examination was conducted for each child to ensure that they had no serious illness and deformity.

### Anthropometry

Weight was measured with a standardized scale and recorded in kilogram to the nearest 0.1kg. Height was measured using a wall-mounted stadiometer and recorded in centimeter to the nearest 0.1cm. The measurements of weight and height follow the standard procedures outlined elsewhere [20, 21]. Body mass index (BMI) was calculated by dividing weight (kg) by the square of height (m). All measurements were conducted in autumn of each year by a fixed group of health professionals. All the health professionals passed a training course before measurements.

### Definition of nutritional status

We used the 2007 World Health Organization (WHO) Growth Chart to calculate Z-score for BMI and height, and then defined the nutritional status for each designated child [22]. As recommended by the WHO Growth Chart, the children aged 24 to 60 months with BMI Z-score greater than 2SD were defined as overweight and those greater than 3SD were defined as obese. The children older than 61 months with BMI Z-score greater than 1SD were defined as overweight and those greater than 2SD were defined as obese [23]. Besides, children whose BMI Z-score was less than 2SD were defined as wasting and those whose Height Z-score was less than -2SD were defined as stunting [24].

### Statistical analysis

The prevalence of wasting, stunting, overweight, and obesity were reported at percentage (%) in each survey year. The differences between girls and boys were tested by Chi-square tests. To acquire the trend information across years, we conducted logistic regressions by using the probability of being overweight, obesity, wasting, and stunning as a function of the survey year and having the survey year as a numeric variable. We used “Maptools” package and the data of boundaries of China from the National Fundamental Geographic Information System of China to make a map showing the geographic location of Luoding city. All analyses were performed by using R software, version 3.3.0 (R Core Team). The *p*<0.05 (two-sided) was defined as statistically significant [25].

## Results

From 2004 to 2013, the population of Luoding increased from 1.10 million to 1.25 million, while the proportion of newborn girls increased from 42.7% to 48.3%. At the same time, the GDP per capital increased from 5.6 thousand yuan to 13.6 thousand yuan (Table 1). A total of 71,059 children’s health examination data were collected in this period, involving 28,779 girls (40.5%) and 42,280 boys (59.5%). The number of children increased from 5,951 in 2004 to 8,749 in 2013. The proportion of girls gradually increased from 35.2% in 2004 to 44.2% in 2013. The average age was 5.1±1.1 years (Table 2).

**Table 1:** The demographic and socioeconomic characteristics of Luoding from 2004 to 2013.

Survey Year	Total population (million)	Birth rate (‰)	Newborn girls	Proportion of newborn girls (%)	Natural increase rate (‰)	GDP per capita (Thousand Yuan)
2004	1.10	11.2	4569	42.7	6.4	5.6
2005	1.11	11.2	6005	48.4	6.7	6.2
2006	1.13	11.0	-	-	6.1	7.1
2007	1.14	10.8	5923	48.5	6.4	6.9
2008	1.16	10.8	5945	48.4	6.1	7.8
2009	1.18	10.7	5968	48.3	6.1	8.3
2010	1.21	10.8	6057	48.5	6.0	9.5
2011	1.22	10.5	6196	48.4	5.8	10.9
2012	1.23	14.0	6339	48.3	8.9	11.9
2013	1.25	12.6	8310	48.3	7.7	13.6

**Table 2:** General characteristics of children.

Survey Year	Number of children	Girls (%) (%)	Age, Mean (SD)(Years) Mean (SD)
2004	5951	35.2	5.2 (1.1)
2005	5907	35.9	5.2 (1.1)
2006	5988	36.9	5.1 (1.1)
2007	6139	38.1	5.1 (1.1)
2008	6413	39.4	5.0 (1.1)
2009	6931	40.7	5.0 (1.1)
2010	7478	42.5	5.1 (1.1)
2011	8695	43.5	5.0 (1.1)
2012	8808	43.9	5.0 (1.1)
2013	8749	44.2	5.2 (1.0)
Total	71059	40.5	5.1 (1.1)

SD: Standard deviation

**Fig 2:**
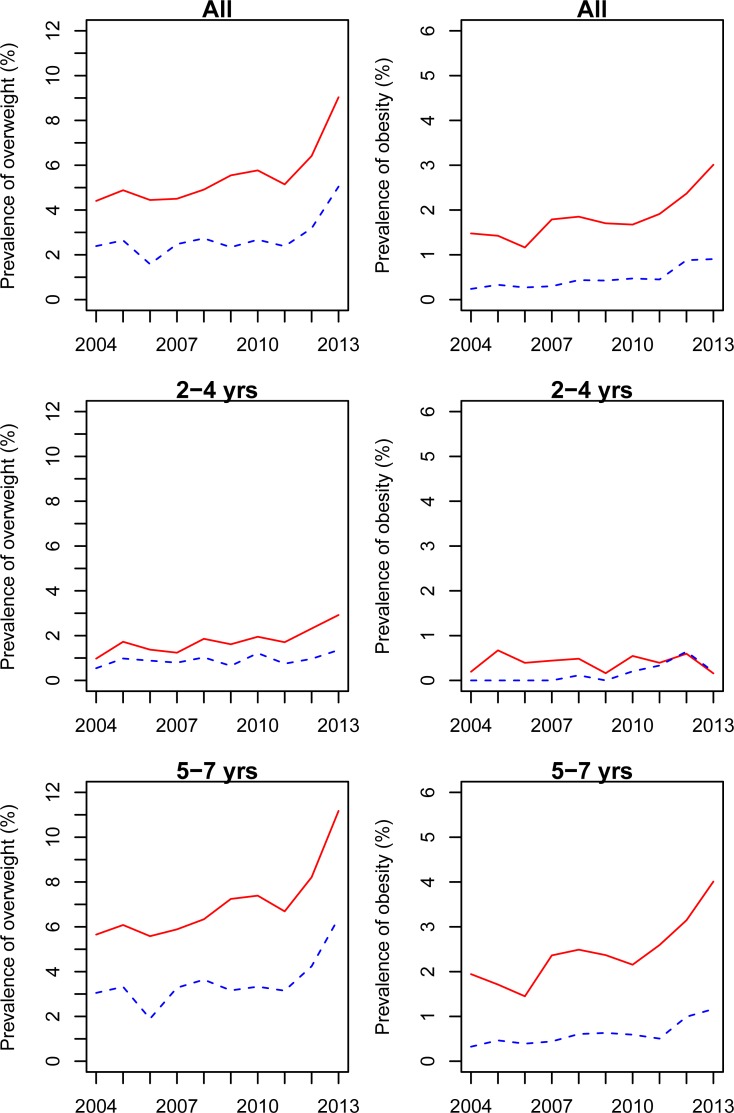
The trends in the prevalence of overweight and obesity among children aged 2 to 7 years from 2004 to 2013.

Table 3 and Fig 2 show the trends in the prevalence of overweight and obesity among pre-school children between 2004 and 2013. The total prevalence of overweight increased from 3.70% in 2004 to 7.27% in 2013 (*p*<0.001 for the trend test across survey years). The risk of overweight increased by 6.9% (95%CI 5.6%, 8.3%) every year. Boys had the higher prevalence of overweight than the girls did each year (*p*<0.05 for Chi-square tests). The prevalence of overweight increased year by year in both boys and girls (*p*<0.05 for the trend test across survey year), and the increase accelerated obviously after 2011, and reaches 9.03% in boys and 5.04% in girls in 2013, respectively.

**Table 3:** The trends in the prevalence of overweight and obesity among children in Luoding from 2004 to 2013.

Survey Year	Total		Boys		Girls	
	Overweight (%)	Obesity (%)	Overweight (%)	Obesity (%)	Overweight (%)	Obesity (%)
2004	3.70	1.04	4.41	1.48	2.39	0.24
2005	4.08	1.03	4.88	1.43	2.64	0.33
2006	3.39	0.84	4.45	1.16	1.58	0.27
2007	3.73	1.22	4.50	1.79	2.48	0.30
2008	4.05	1.29	4.91	1.85	2.73	0.44
2009	4.24	1.18	5.55	1.70	2.34	0.43
2010	4.45	1.16	5.77	1.67	2.67	0.47
2011	3.94	1.28	5.15	1.91	2.38	0.45
2012	5.00	1.71	6.41	2.37	3.18	0.88
2013	7.27	2.08	9.03	3.01	5.04	0.91

The total prevalence of obesity increased from 1.04% in 2004 to 2.08% in 2013 (*p* = 0.012 for the trend test across survey year). The risk of obesity increased by 8.4% (95%CI 5.9%, 11.0%) every year. Boys had the higher prevalence of obesity than the girls did each year (*p*<0.05 for Chi-square tests). The prevalence of obesity also rose in both boys and girls (*p*<0.05 for the trend test across survey year), and reached 3.01% in boys and 0.91% in girls in 2013, respectively. The upward trend accelerated obviously after 2011 in both girls and boys.

For age-specific trend, older children (aged 5 to 7 years) had higher growth rate of overweight and obesity than younger children (aged 2 to 4 years) did. We found the prevalence of overweight among children aged 2 to 4 years and the prevalence of overweight and obesity among children aged 5 to 7 years kept increasing from 2004 to 2013.

**Fig 3:**
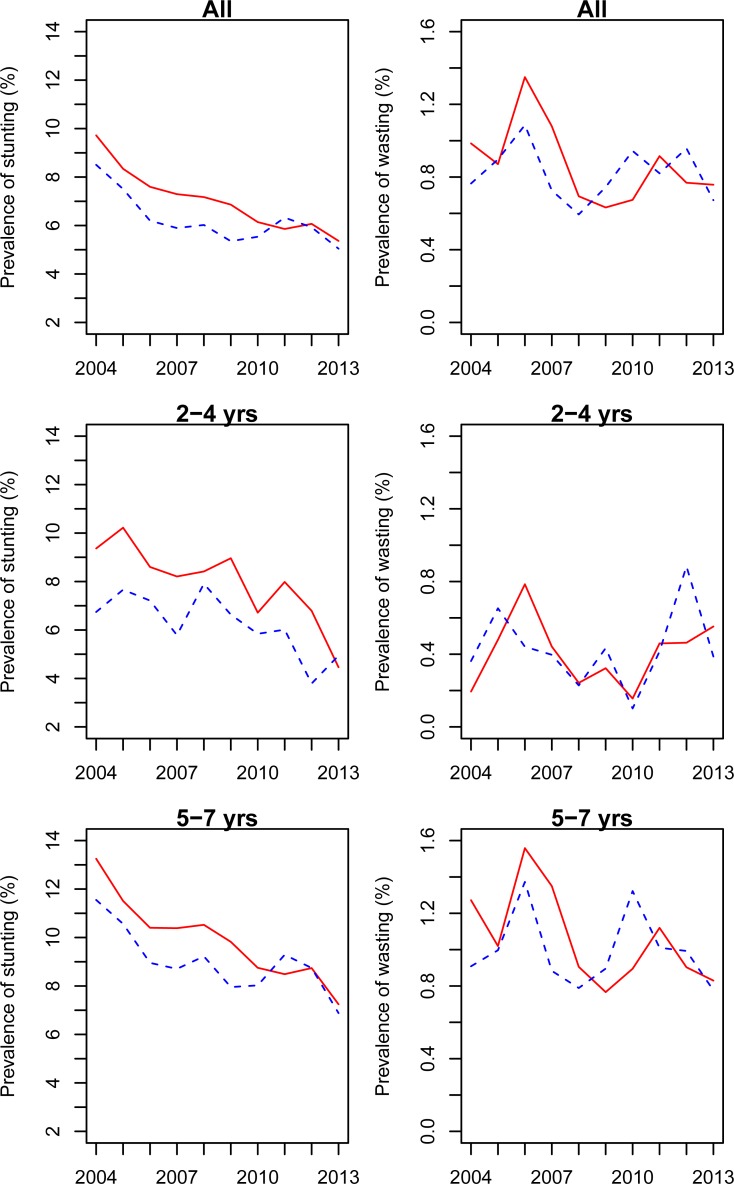
The trends in the prevalence of stunting and wasting among children aged 2 to 7 years from 2004 to 2013.

Table 4 and Fig 3 indicate the trends in the prevalence of stunting and wasting among pre-school children between 2004 and 2013. The total prevalence of wasting declined from 0.91% in 2004 to 0.72% in 2013 (*p* = 0.014 for the trend test across survey year). The risk of wasting decreased by 2.8% (95%CI 0.1%, 5.5%) every year. The prevalence of wasting was slightly decreasing between 2004 and 2013 in both boys and girls (*p*<0.05 for trend test across survey year), and reached 0.76% in boys and 0.67% in girls in 2013, respectively.

**Table 4:** The trends in the prevalence of stunting and wasting among children in Luoding from 2004 to 2013.

Survey Years	Total		Boys		Girls	
	Stunting (%)	Wasting (%)	Stunting (%)	Wasting (%)	Stunting (%)	Wasting (%)
2004	9.29	0.91	9.72	0.98	8.50	0.76
2005	8.04	0.88	8.34	0.87	7.51	0.90
2006	7.08	1.25	7.60	1.35	6.20	1.09
2007	6.76	0.94	7.29	1.08	5.89	0.73
2008	6.72	0.65	7.17	0.69	6.02	0.59
2009	6.25	0.68	6.86	0.63	5.35	0.74
2010	5.88	0.79	6.14	0.67	5.54	0.94
2011	6.06	0.87	5.86	0.92	6.33	0.82
2012	6.01	0.85	6.07	0.77	5.93	0.96
2013	5.22	0.72	5.37	0.76	5.04	0.67

The total prevalence of stunting dropped from 9.29% in 2004 to 5.22% in 2013 (*p* = 0.005 for the trend test across survey year). The risk of stunting decreased by 5.4% (95%CI 4.5%, 6.4%) every year. The prevalence of stunting was decreasing between 2004 and 2013 in both boys and girls (*p*<0.05 for the trend test across survey year), and reached 5.37% in boys and 5.04% in girls in 2013, respectively.

## Discussion

The present study illustrates that in the last decade, the prevalence of overweight and obesity increased and the prevalence of wasting and stunting decreased in Luoding city, China. While the prevalence of overweight and obesity reached 7.27% and 2.08%, there were still 0.72% and 5.22% of children being wasting and stunting in 2013. The results indicate a double burden of overnutrition and undernutrition in Luoding, China. Comprehensive interventions should be implemented in similar lower-middle-income areas to control overweight and obesity and improve stunting and wasting.

Some studies have presented the similar information about pre-school children’s overnutrition. A cross-sectional survey from 9 Chinese cities reported that the prevalence of obesity, defined with a weight-for-height ≥ 120% of the National Center for Health Statistics/World Health Organization reference, increased from 0.9% in 1986 to 3.44% in 2006 in children aged 0 to 7 years [19]. Another study in Tianjin, a metropolis city in economically developed area of China, showed that the prevalence of obesity on children aged 5 to 6 years, which was defined by a BMI Z-score > 2SD in the WHO Growth Chart reference, increased significantly from 8.8% in 2006 to 10.1% in 2010. They also reported that between 2010 and 2014, the prevalence of obesity kept constant around 10.1% [18]. Various studies in the western countries also reported the increasing prevalence of obesity among pre-school children [23, 26, 27]. These findings confirmed that childhood obesity increased continuously in the last decade. In some high-income cities (e.g., Tianjin), the prevalence stabilized in recent year due to the increasing awareness of the risk of childhood obesity in the families, schools and society and more obesity intervention programs. However, our study in Luoding showed an increasing trend of obesity in pre-school children, suggesting that it would be more effective to take early interventions to reverse the increasing trend in specific people and areas.

Even the prevalence of undernutrition has been decreasing since last decade, our study indicates that there were still 0.72% and 5.22% of children being wasting and stunting in 2013. A national survey based on the Chinese Nutrition and Health Surveillance shows that there was a decline of wasting and stunting (defined as a Height Z-score < -2SD or BMI Z-score < -2SD of the WHO Growth Chart reference) between 2002 and 2013 among children under 5, but the overall prevalence of wasting and stunting of Chinese children under 5 were 1.9% and 8.1% in 2013. The prevalence of stunting was higher than that of wasting. Stunting was related to increased risk for metabolic diseases such as obesity, cardiovascular disease, and diabetes in adulthood [11]. These findings indicated that although childhood undernutrition was improved dramatically in the last decade, the certain proportion of undernutrition in pre-school children is still a challenge to public health in the low-middle-income areas, and there is an urgent need to develop effective health interventions or policies.

As far as we know, there are few studies on “double burden” of undernutrition and overnutrition among pre-school children in lower-middle-income areas [9, 16]. The findings in this study provide meaningful evidences to guide future public health interventions for these children. With the increase of family income, dietary patterns with higher energy, fat and animal-sourced foods have replaced Chinese traditional dietary patterns in many areas [13, 28, 29]. This change is closely related to the increasing prevalence of overnutrition among children in these areas [13, 28]. More sedentary behaviors, less physical activities and shorter sleep also contribute to the increasing prevalence of overweight and obesity [30, 31]. However, due to the disparities of social and economic development in China, nutritional insufficiencies still affect many children in lower-middle-income areas. A previous study shows that children whose family within the poorest quintile had 2.47 times (range 1.00–7.64) higher prevalence of stunting than those within the richest quintile [5]. The present study also shows that stunting coexisted with obesity. The shortage of health awareness, nutrient inadequacies [10], and preference of energy-dense foods (e.g., fried food, puffed food) could be the reasons of this phenomenon. Therefore, more attention should be paid simultaneously to undernutrition when we conduct interventions for overnutrition in these areas.

Many studies argue that early childhood malnutrition (both stunting and overweight) could contribute to later nutrition-related diseases [11]. In addition, dietary and physical activity habits are formed in pre-school years, which would be more improvable than in later childhood [8, 32]. Developing effective early interventions would be an important means for controlling the overnutrition epidemic and reducing the prevalence of undernutrition for pre-school children. In lower-middle-income areas such as Luoding city, comprehensive health policies including lifestyle improvement, food selection, nutritional supplementation and physical activity promotion should be implemented among pre-school children, especially the group aged 5 to 7 year, as we found they had higher prevalence of overweight and obesity.

There are several strengths in this study. First, this study is based on the large panel data of pre-school children from 23 kindergartens from 2004 to 2013, including children’s weight and height, which are measured by trained health professionals. Second, the children focused in this study are from Luoding, a typically lower-middle-income city of China, which could fill the evidence gap for similar areas.

However, there are some limitations in this study. First, the trend in the nutritional status has not been investigated longitudinally without the cohort data of the same children. As the survey was based on kindergartens, there is the possibility that some children are represented up to 3 times of the data (there are three grades in the kindergartens), the surveys in different years might not be completely independent. However, we conducted sensitivity analyses in the subset, which selected participants with same age from each survey, and obtained similar results with previously mentioned. Second, the detailed information of individual socioeconomic status, behaviors, parents’ knowledge and attitude, etc. were unavailable in this study, which impedes us to determine the reasons for the trends in the prevalence of undernutrition and overnutrition among preschool children and further studies are needed.

## Conclusions

In the past ten years, the prevalence of overnutrition (both overweight an obesity) showed tendency to ascend, meanwhile, the prevalence of undernutrition (both wasting and stunting) decreased in pre-school children aged 2 to 7 in Luoding city, China. There was a double burden of undernutrition and overnutrition. The results of the present study suggest that comprehensive nutritional interventions should be implemented for pre-school children in lower-middle-income areas.
